# Supplementary material for: Pulcherriminic acid modulates iron availability and protects against oxidative stress during microbial interactions
Source: Nat Commun. 2023 May 3;14:2536. doi: 10.1038/s41467-023-38222-0 (PMC10156857; doi:10.1038/s41467-023-38222-0)
Supplement: Supplementary file 1 — Supplementary information [file 41467_2023_38222_MOESM1_ESM.pdf]

Supplementary Methods

Pulcherriminic acid synthesis – General details

All non-aqueous reactions involving air or moisture sensitive compounds were run under an inert atmosphere (nitrogen or argon) with rigid exclusion of moisture from reagents and glassware using standard techniques. All glassware was stored in the oven and/or was flame dried prior to use under an inert atmosphere of gas. The solvents and chemicals used for the procedure were acquired either at Sigma-Aldrich or Fisher Canada (distributing Acros, TCI, and Alfa Aesar chemicals in Canada). Anhydrous solvents were obtained either by distillation over sodium (THF, ether), over calcium hydride (CH_2Cl_2). Analytical thin-layer chromatography (TLC) was performed on precoated, glass-backed silica gel (Merck 60 F_{254}). Visualization of the developed chromatogram was performed by UV absorbance, aqueous cerium molybdate, ethanolic phosphomolybdic acid, iodine, or aqueous potassium permanganate. Flash column chromatography was performed using 230-400 mesh silica (EM Science or Silicycle) of the indicated solvent system according to standard technique. Chemical purity was assessed by analysis of ^{1}H and ^{13}C NMR spectra. Infrared spectra were taken on a Perkin Elmer Spectrum One FTIR and are reported in reciprocal centimeters (cm^{-1}). Nuclear magnetic resonance spectra (^{1}H, ^{13}C, DEPT, COSY, HMQC) were recorded either on a Bruker Avance III HD 300 or Varian Mercury+ 400 spectrometer. Chemical shifts for ^{1}H NMR spectra are recorded in parts per million from tetramethylsilane with the solvent resonance as the internal standard. Data are reported as follows: chemical shift, multiplicity (s = singlet, d = doublet, t = triplet, q = quartet, qn = quintet, sext = sextuplet, non = nonet, m = multiplet and br = broad), coupling constant in Hz, integration. Chemical shifts for ^{13}C NMR spectra are recorded in parts per million from tetramethylsilane with the solvent resonance as the internal standard. When ambiguous, proton and carbon assignments were established using COSY, NOESY, HMQC and DEPT experiments. High resolution mass spectra were performed at Université de Sherbrooke. Melting points were obtained on a Buchi melting point apparatus and are uncorrected.

3,6-diisobutylpiperazine-2,5-dione (1)

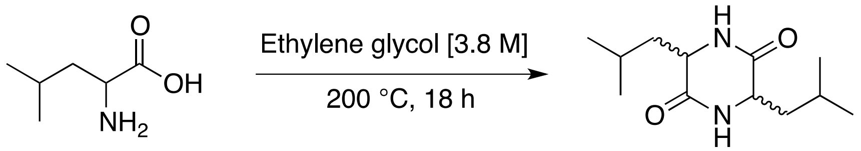

1

Method based on a modified procedure[1]. In a sealed tube, flame dried and under argon, (*L*)-leucine (3.28 g, 25 mmol) was put in suspension in Ethylene Glycol (6.58 ml, [3.8 M]). The white bread-like mixture was heated at 200°C for 18 hours and became a brown/copper colored solution with agitation. After cooling to room temperature, the orange solid was washed with EtOAc (75 ml) then *i*-PrOH (10 ml), then dried *in-vacuo* to obtain an inconsequential mixture of isomers of 3,6-diisobutylpiperazine-2,5-dione (**1**) (1.72 g, 61 %) as a white solid, which could be used directly for the next step. **^{1}H NMR** (400 MHz, $CDCl_3$) δ (ppm) 6.17–6.11 (m, 2H), 4.02–3.94 (m, 2H), 1.93–1.73 (m, 4H), 1.70–1.58 (m, 2H), 1.02–0.94 (m, 12H). **^{13}C NMR** (100 MHz, $CDCl_3$) δ (ppm) 168.98,

168.85, 53.47, 53.30, 43.46, 42.23, 24.45, 24.37, 23.40, 23.31, 21.40, 21.25. NMR analysis is consistent with the known compound[2].

3-chloro-2,5-diisobutylpyrazine (2) and 2,5-dichloro-3,6-diisobutylpyrazine (3)

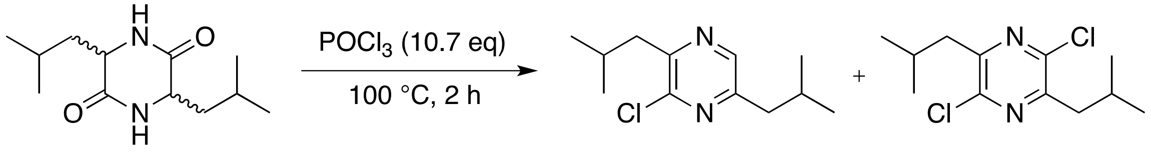

1 **2** **3**

<u>*Procedure for chlorination*</u>*, based on a modified procedure*[1]*.* In a sealed tube, flame dried and under argon, 3,6-diisobutylpiperazine-2,5-dione (**1**) (905 mg, 4 mmol) was solubilized in Phosphorus(V) Oxychloride (4 ml, 42.8 mmol), then heated at 100°C and stirred for 2 hours. After the indicated time, the red mixture was cooled to room temperature, then poured slowly in stirred ice-cooled water (50 ml). After addition and 15 min of stirring, CH_2Cl_2 (25 ml) was added, then the organic layer was extracted twice and washed with $NaHCO_3$ (sat.) (2x 25 ml). The organic layer was dried with $MgSO_4$, filtered and concentrated. The products were separated by flash chromatography on silica, using a gradient from 1% to 2% Et_2O / hexanes.

3-chloro-2,5-diisobutylpyrazine (2), pale yellow oil (542 mg, 60 %): **^{1}H NMR** ^{1}H NMR (400 MHz, Chloroform-*d*) δ (ppm) 8.23 (s, 1H), 2.80 (d, *J* = 7.2 Hz, 2H), 2.61 (d, *J* = 7.2 Hz, 2H), 2.21 (non, *J* = 6.8 Hz, 1H), 2.10 (non, *J* = 6.8 Hz, 1H), 0.96 (d, *J* = 6.7 Hz, 6H), 0.93 (d, *J* = 6.7 Hz, 6H); **^{13}C NMR** (100 MHz, $CDCl_3$) δ (ppm) 154.22, 152.52, 148.26, 141.88, 43.60, 43.26, 28.95, 28.05, 22.51, 22.36; ***R_f*** (1% Et_2O / hexanes) : 0.05. NMR analysis is consistent with the known compound[3].

2,5-dichloro-3,6-diisobutylpyrazine (3), clear oil (150 mg, 14 %): **^{1}H NMR** (400 MHz, Chloroform-*d*) δ (ppm) 2.76 (d, *J* = 7.2 Hz, 4H), 2.21 (non, *J* = 6.8 Hz, 2H), 0.96 (d, *J* = 6.7 Hz, 12H); **^{13}C NMR** (100 MHz, $CDCl_3$) δ (ppm) 152.69, 145.99, 42.82, 28.24, 22.51; **IR** (Neat) ν (cm^{-1}) 2950, 2925, 2875, 1475, 1400, 1325, 1100; ***R_f*** (1% Et_2O / hexanes) : 0.30; **HRMS (ESI+)** (m/z) calcd for $C_{12}H_{18}Cl_2N_2$ $[M+Na]^+$: 283.0739, found : 283.0745.

2,5-dichloro-3,6-diisobutylpyrazine (3)

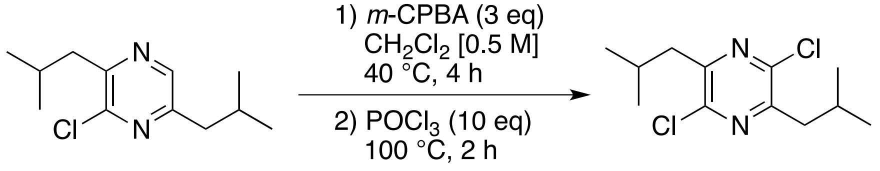

2 **3**

Method based on a modified procedure[1]*.* In a vial, 3-chloro-2,5-diisobutylpyrazine (**2**) (188 mg, 829 µmol) was solubilised in CH_2Cl_2 (1.66 ml, [0.5M]), then *m*-CPBA (429 mg, 2.49 mmol) was added. The mixture was heated at 40°C and stirred for 4 hours. After the indicated time and cooling to room temperature, EtOAc (20 ml) was added, and the organic layer was washed with Na_2SO_3 (10 % in water, 3x 20ml), then $NaHCO_3$ (sat. 3x 20ml). The organic layer was dried with $MgSO_4$, filtered and concentrated *in-vacuo*.

Without purification, the *procedure for chlorination* was performed to obtain 2,5-dichloro-3,6-diisobutylpyrazine (**3**) (84.6 mg, 39 %)

2,5-dichloro-3,6-diisobutylpyrazine 1,4-dioxide (4)

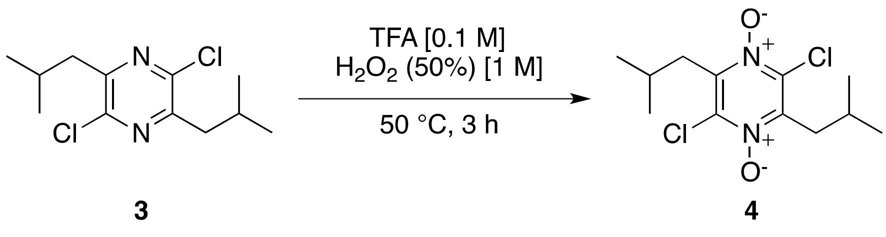

In a round bottom flask, 2,5-dichloro-3,6-diisobutylpyrazine (**3**) (360 mg, 1.38 mmol) was solubilized in THF (13.8 ml, [0.1 M]). The solution was cooled to 0°C, then H_2O_2 (50% in water, 1.38 ml, [1M]) was added dropwise. After addition, the mixture was heated at 50°C and stirred for 3 hours. After cooling to room temperature, CH_2Cl_2 (10 ml) was added, then the mixture was poured slowly in saturated Na_2CO_3 with ice (*c.a.* 30 ml). Na_2SO_3 (10 % in water, 20 ml) was added, and the mixture was stirred 15 min at room temperature. The organic layer was extracted using CH_2Cl_2 (2x 25 ml), then dried over $MgSO_4$, filtered and concentrated. The product was purified using flash chromatography on silica, with 1% MeOH in CH_2Cl_2 as the eluant. 2,5-dichloro-3,6-diisobutylpyrazine 1,4-dioxide (**4**) was obtained as a yellow solid (260 mg, 64 %). **mp** 191°C; **^{1}H NMR** (400 MHz, $CDCl_3$) δ (ppm) 3.03 (d, *J* = 7.3 Hz, 4H), 2.34 (non, *J* = 6.8 Hz, 2H), 1.02 (d, *J* = 6.7 Hz, 12H); **^{13}C NMR** (100 MHz, $CDCl_3$) δ (ppm) 146.22, 139.81, 37.97, 26.35, 22.72; **IR** (Neat) ν (cm^{-1}) 2950, 2925, 2850, 1475, 1300, 1225, 1125, 1000; ***R_f*** (1% MeOH / DCM) 0.45; **HRMS (ESI+)** (m/z) calcd for $C_{12}H_{18}Cl_2N_2O_2$ $[M+Na]^+$: 315.0638, found : 315.0645.

2,5-dihydroxy-3,6-diisobutylpyrazine 1,4-dioxide (Pulcherriminic acid) (5)

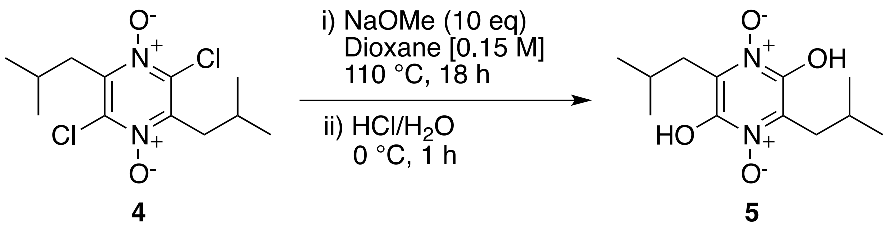

In a sealed tube, flame dried and under argon, sodium methoxide (326 µL, 4.88 mmol) was weighted (in a glove box) then, under argon flow, 2,5-dichloro-3,6-diisobutylpyrazine 1,4-dioxide (**4**) (143 mg, 488 µmol) was added. Finally dry 1,4-dioxane (3.26 ml, [0.15M]) was added to the solid mixture. The mixture was heated at 110°C and stirred for 18 hours. After cooling, the solvent was evaporated, and the product was dissolved in water (20 ml). The aqueous layer was washed with ether (2x 20ml), then the organic layer was removed. The aqueous layer was cooled to 0°C, then brought to pH 1 with concentrated HCl. The mixture was allowed to return to room temperature while stirring for 1h, then cooled back to 0 °C before filtration. The product is purified by trituration with pentane and Et_2O to give

pulcherriminic acid (**5**) (49.9 mg, 40%) as a white solid. **^{1}H NMR** (400 MHz, DMSO-d_6) δ (ppm) 2.68 (d, *J* = 7.3 Hz, 4H), 2.17-2.06 (m, 2H), 0.89 (d, *J* = 6.7 Hz, 12H). **^{13}C NMR** (100 MHz, DMSO-d_6) δ (ppm) 145.50, 129.61, 33.39, 26.20, 22.37; **IR** (Neat) ν (cm^{-1}) 3250-2000(br) 2957, 2868, 1654, 1578, 1511, 1466, 1394, 1220, 1153, 1067, 998, 967; **HRMS (ESI-)** (m/z) calcd for $C_{12}H_{19}N_2O_4$ $[M-H]^-$: 255.1350, found : 255.1352.

3,6-diisobutylpiperazine-2,5-dione (1)

1H NMR (400MHz, CDCl3)

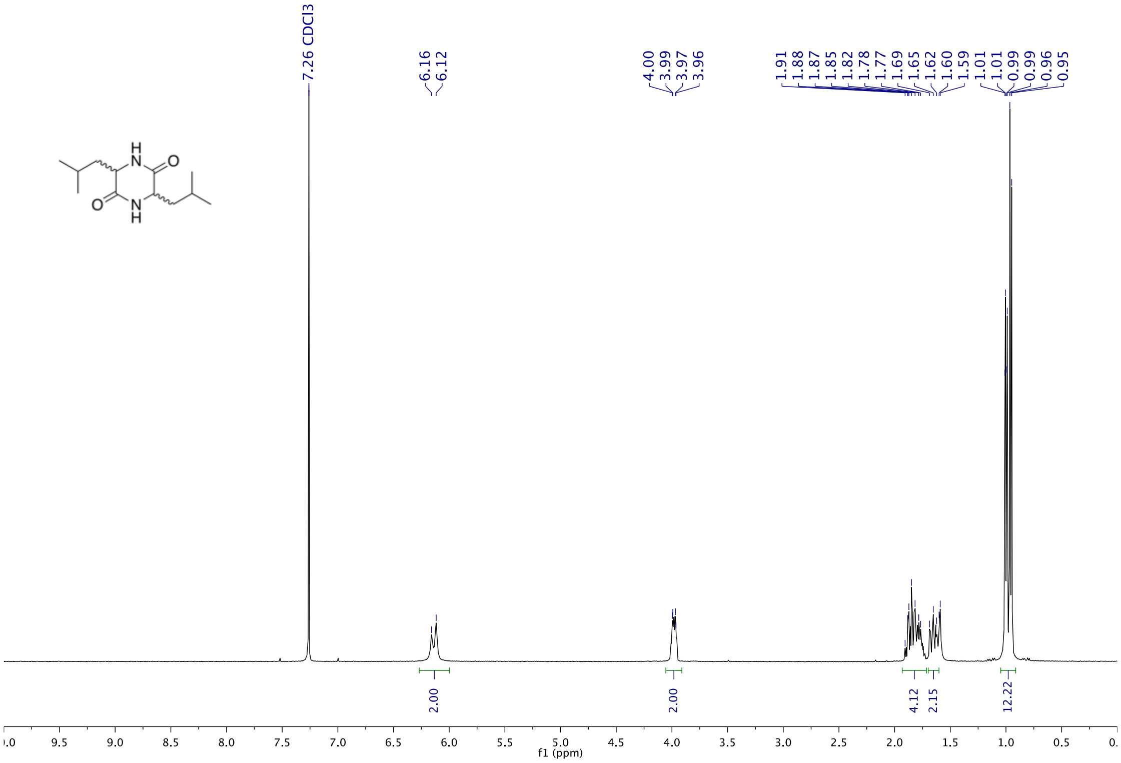

13C NMR (100MHz, CDCl3)

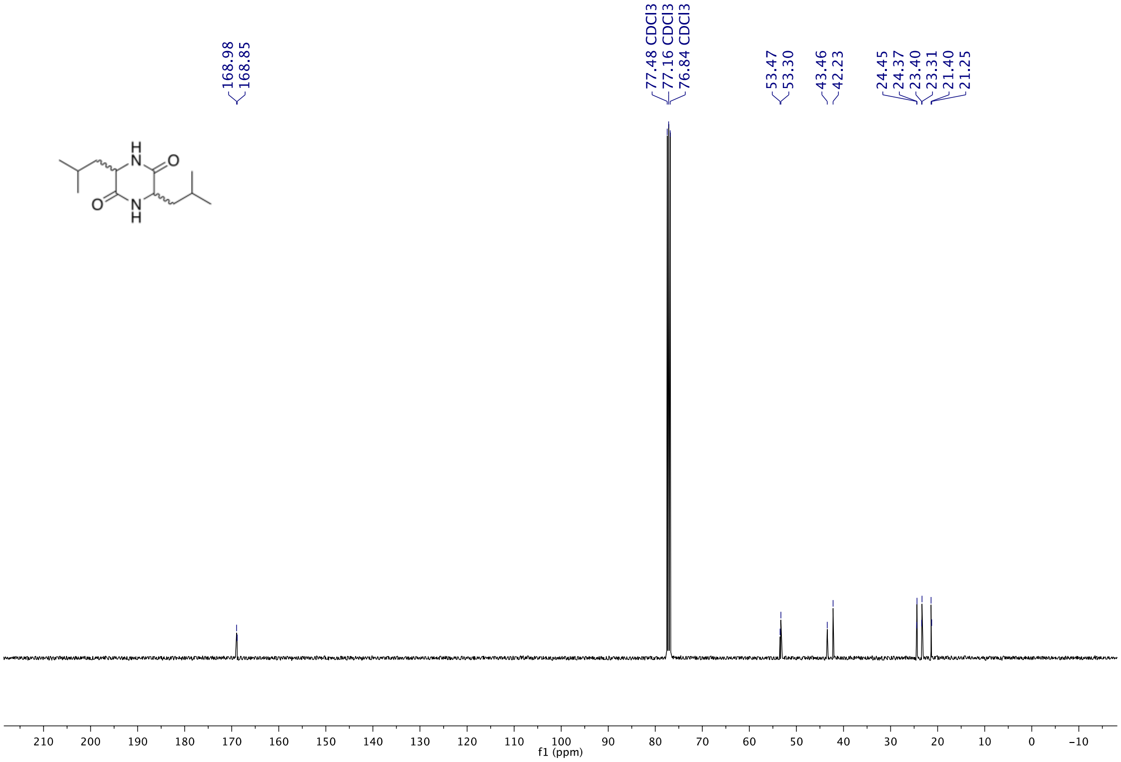

3-chloro-2,5-diisobutylpyrazine (2)

1H NMR (400MHz, CDCl3)

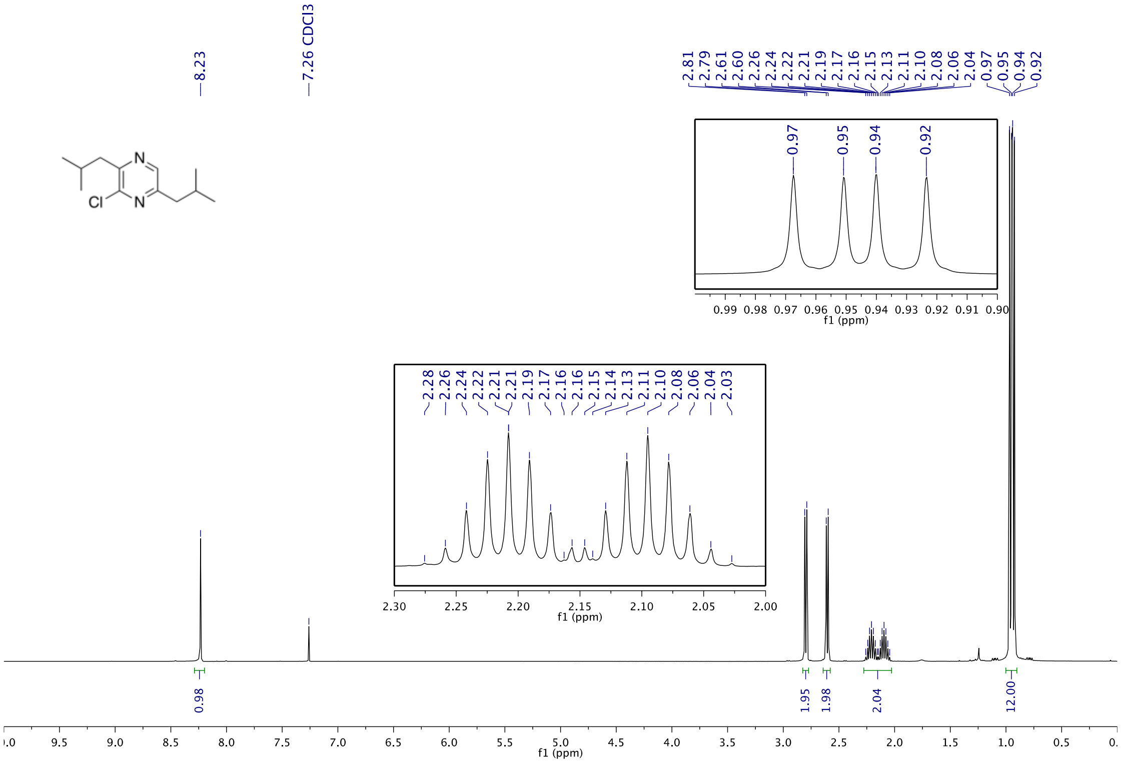

13C NMR (100MHz, CDCl3)

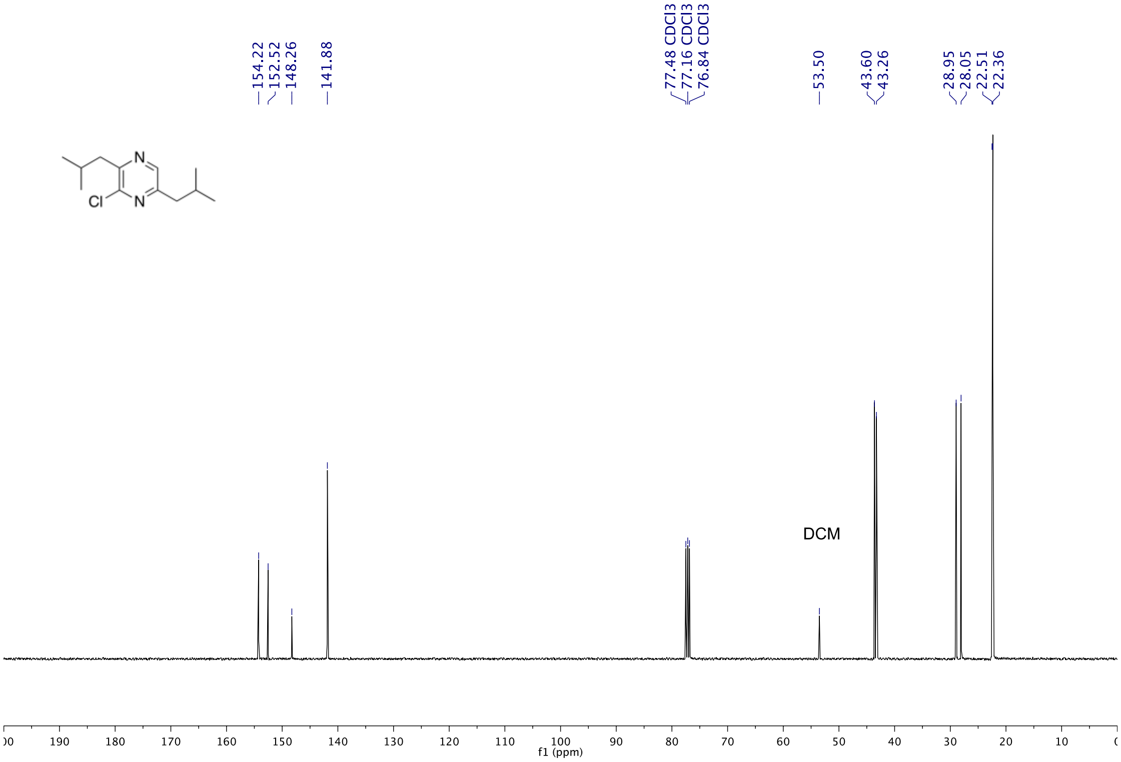

2,5-dichloro-3,6-diisobutylpyrazine (3)

1H NMR (400MHz, CDCl3)

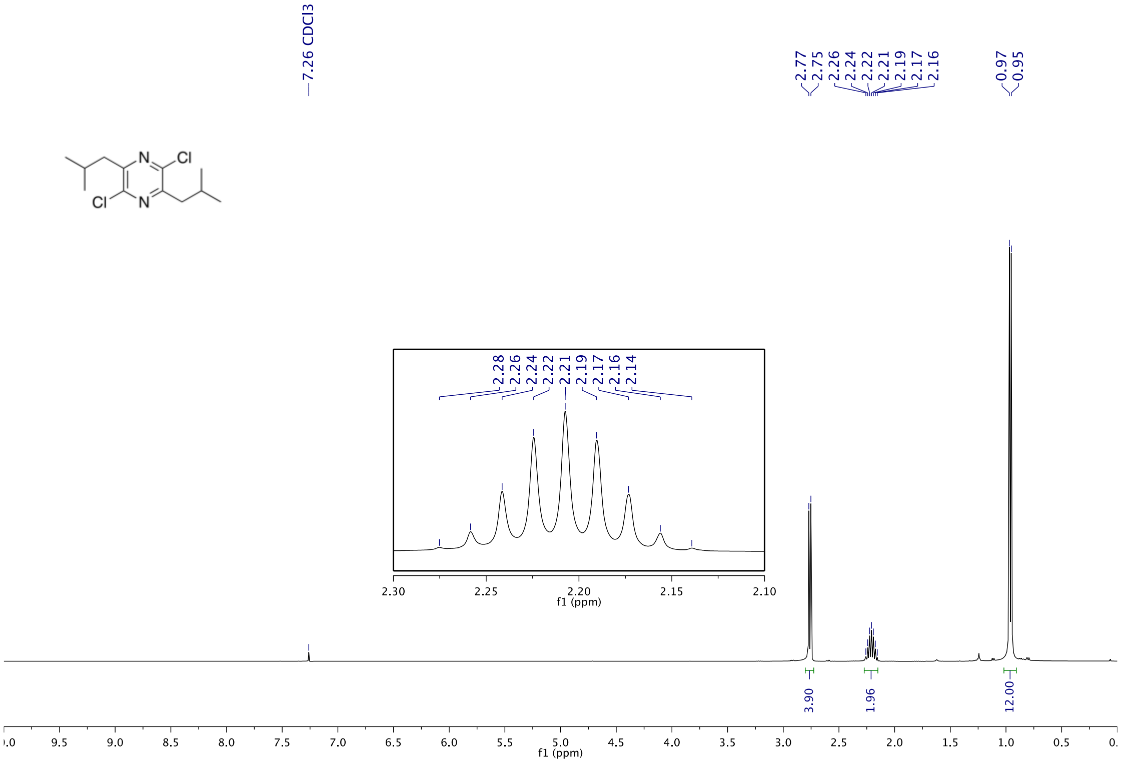

13C NMR (100MHz, CDCl3)

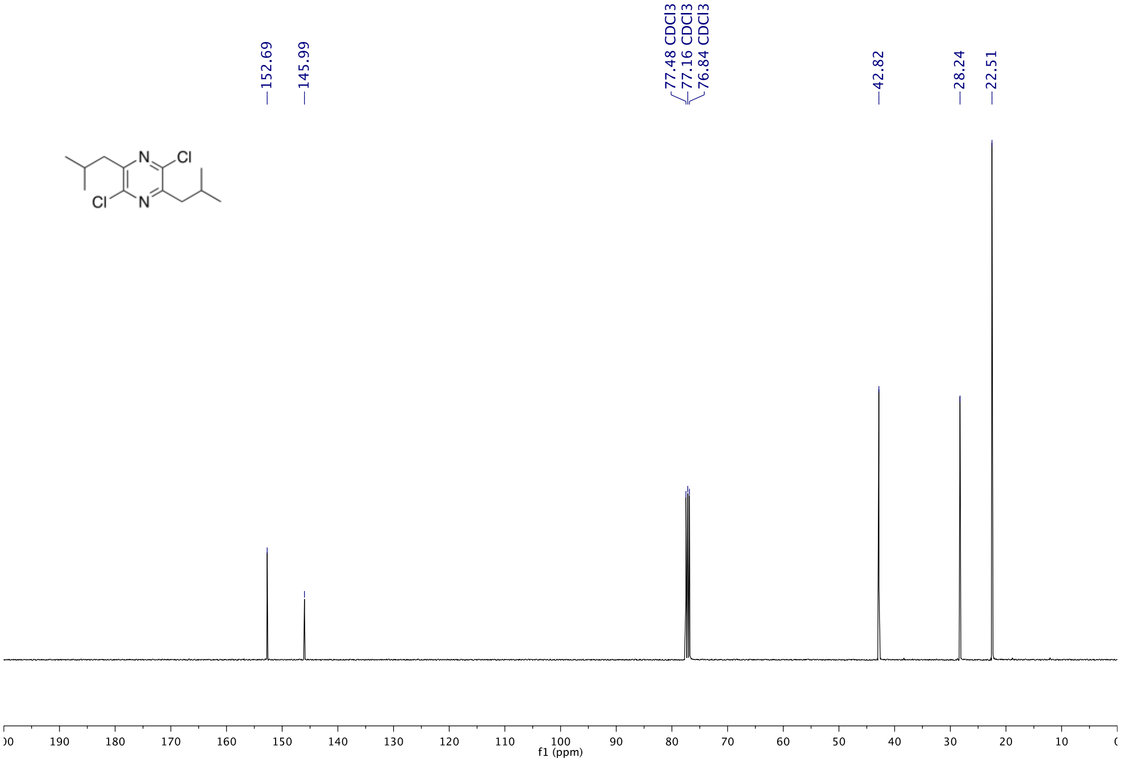

2,5-dichloro-3,6-diisobutylpyrazine 1,4-dioxide (4)

1H NMR (400MHz, CDCl3)

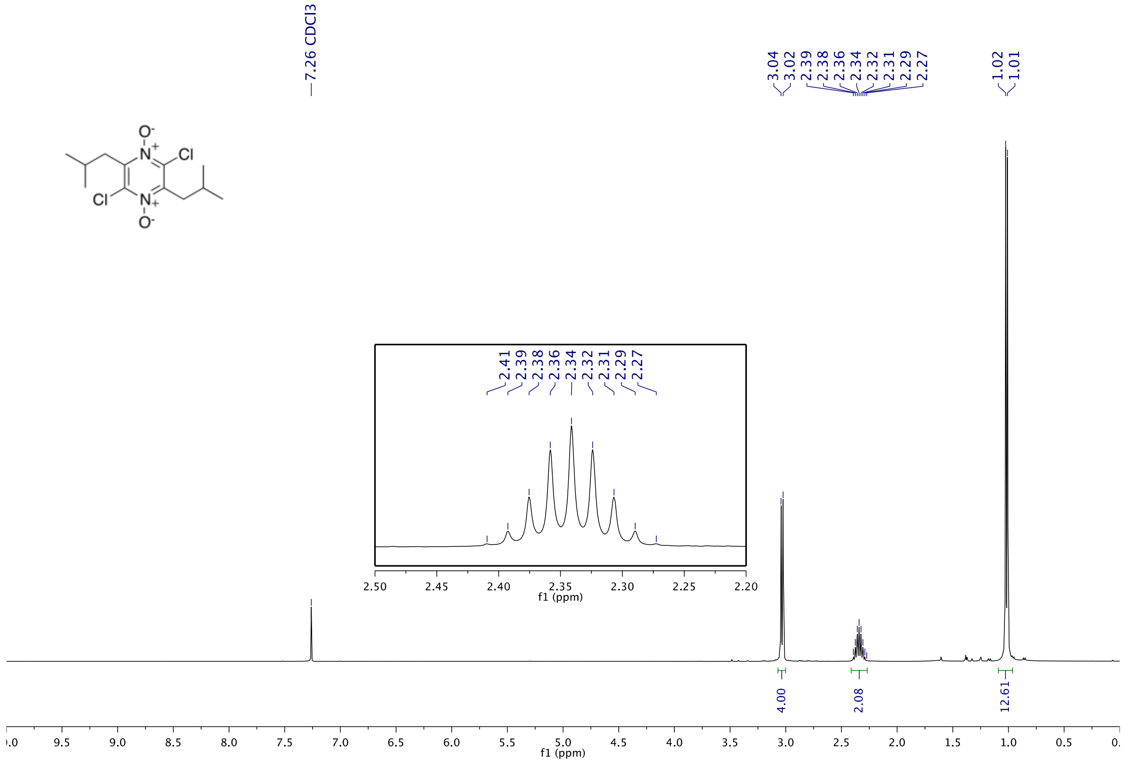

13C NMR (100MHz, CDCl3)

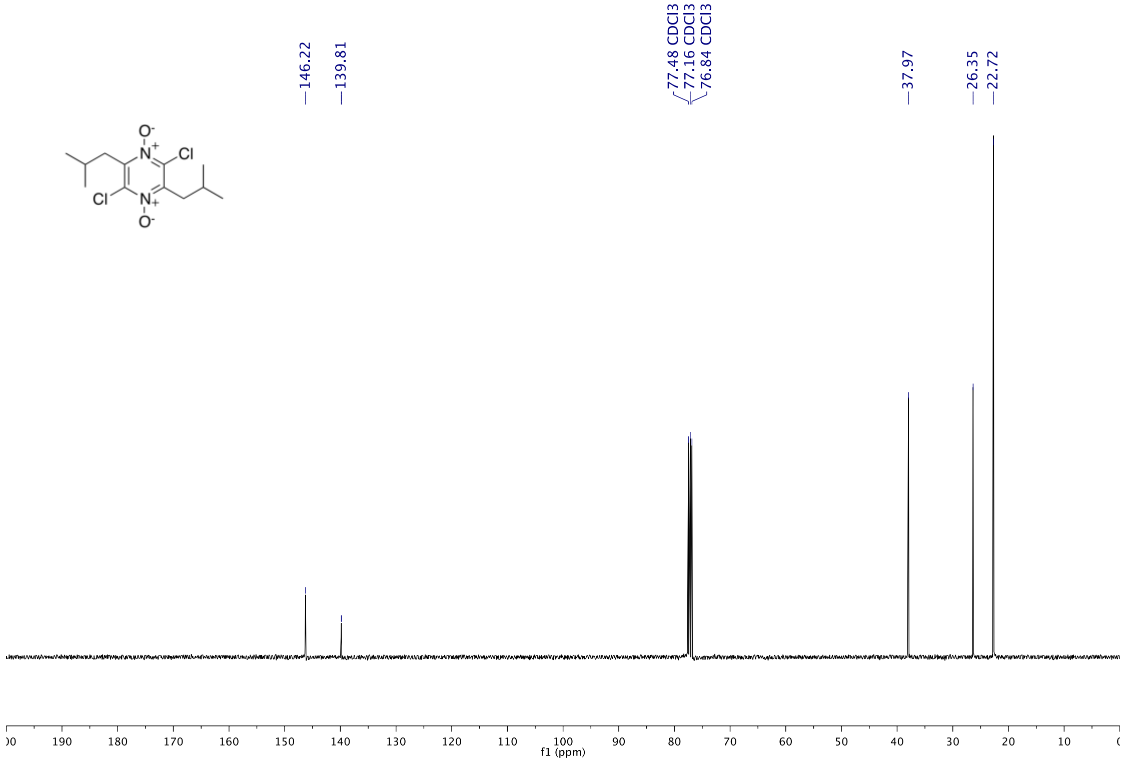

2,5-dihydroxy-3,6-diisobutylpyrazine 1,4-dioxide (pulcherriminic acid) (5)

1H NMR (400MHz, DMSO-d6)

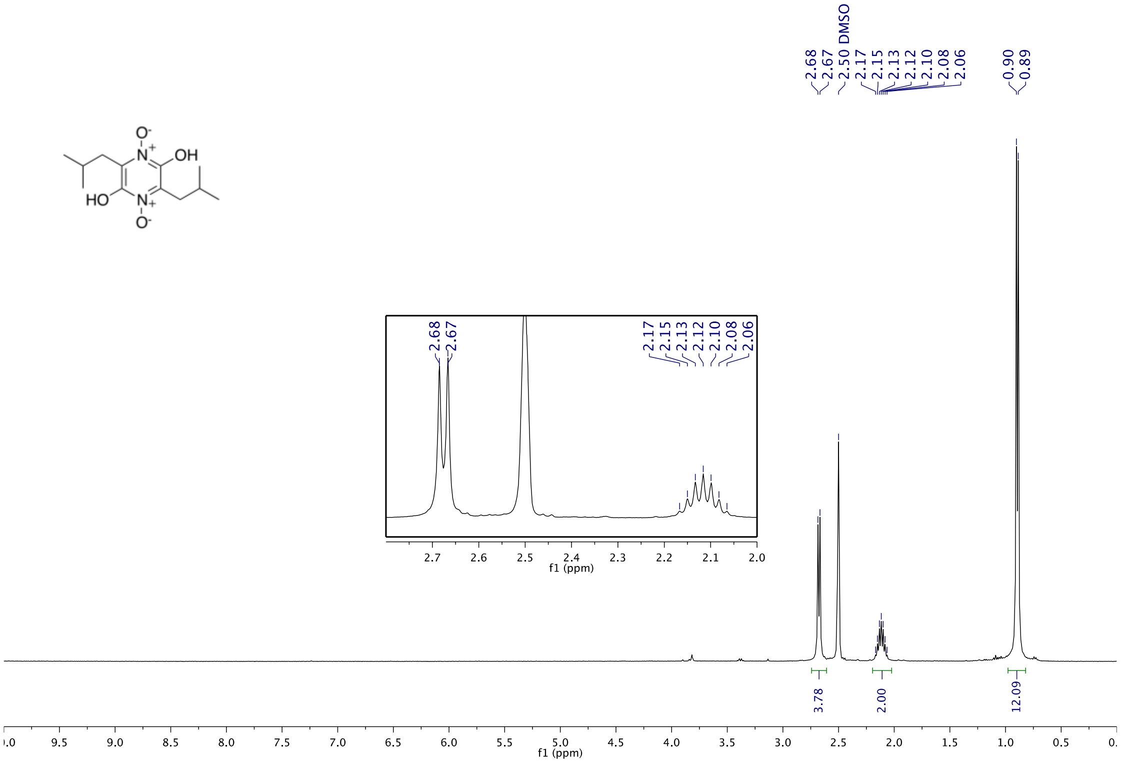

13C NMR (100MHz, DMSO-d6)

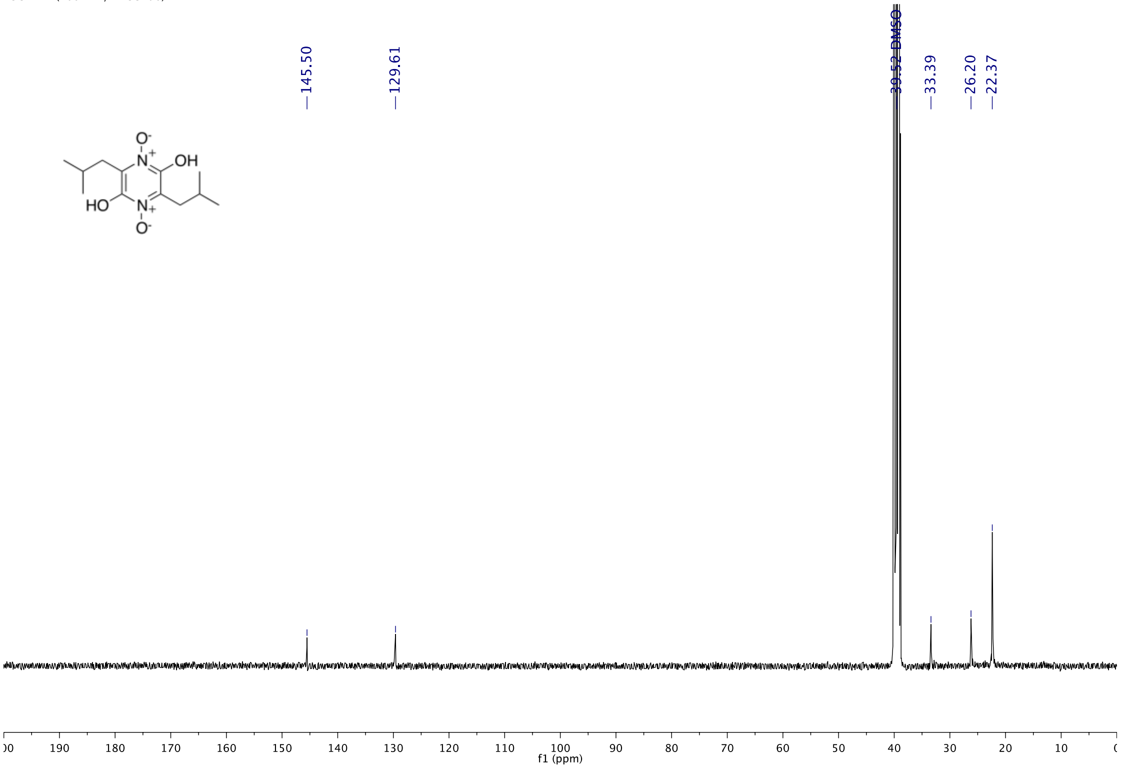

Supplementary Tables S1 – S3

Table S1: Strains used in this study.

Strain	Genotype	Reference
NCIB 3610	WT / undomesticated	Lab stock
168	Domesticated	Lab stock
Escherichia coli NEB5α	For molecular cloning	Lab stock
BKK35070	168 *yvmC*::kan	BGSC[4]
VCL97	3610 *yvmC*::kan	This study
BKK35080	168 *pchR*::kan	BGSC[4]
PB755	3610 *pchR*::kan	Lab stock
BKK31960	168 *dhbF*::kan	BGSC[4]
VCL109	3610 *dhbF*::kan	This study
VCL100	3610 *amyE*::P_{yvmC}-*yfp*	This study
PB503	3610 *dhbA-F*::erm	Lab stock
VCL111	3610 *amyE*::P_{dhbA}-*lacZ*	This study
VCL112	3610 *yvmC*::kan *amyE*::P_{dhbA}-*lacZ*	This study
VCL113	3610 *pchR*::kan *amyE*::P_{dhbA}-*lacZ*	This study
VCL118	3610 *yvmC*::kan *amyE*::P_{yvmC}-*yvmC-cypX*	This study
Pseudomonas fluorescens	WCS365	Lab stock
Pseudomonas capeferrum	WCS358	Lab stock
Pseudomonas protegens	Pf-5	Lab stock
Pseudomonas protegens	*phlD*::tet	Lab stock

Antibiotics resistance abbreviations: Kanamycin (kan), tetracycline (tet), erythromycin (erm)

Table S2: Regression parameters

**Analytes**	**Concentration** (ng/mL)	**Linear equation** Y=a X+b	R^2
Bacillibactin (BB)	0.01-2.5	Y= 10410 X – 1104	0.985
Pulcherriminic acid (PA)	0.1-10	Y= 595.77 X + 282.4	0.995

Table S3: Limit of quantification

**Analytes**	**Recovery (%)**	**RSD (%)**	**LOD (ng/mL)**	**LOQ (ng/mL)**
Bacillibactin (BB)	72.1	7.01	0.017	0.029
Pulcherriminic acid (PA)	83.9	8.59	0.145	0.211

Supplementary Figures S1 – S9

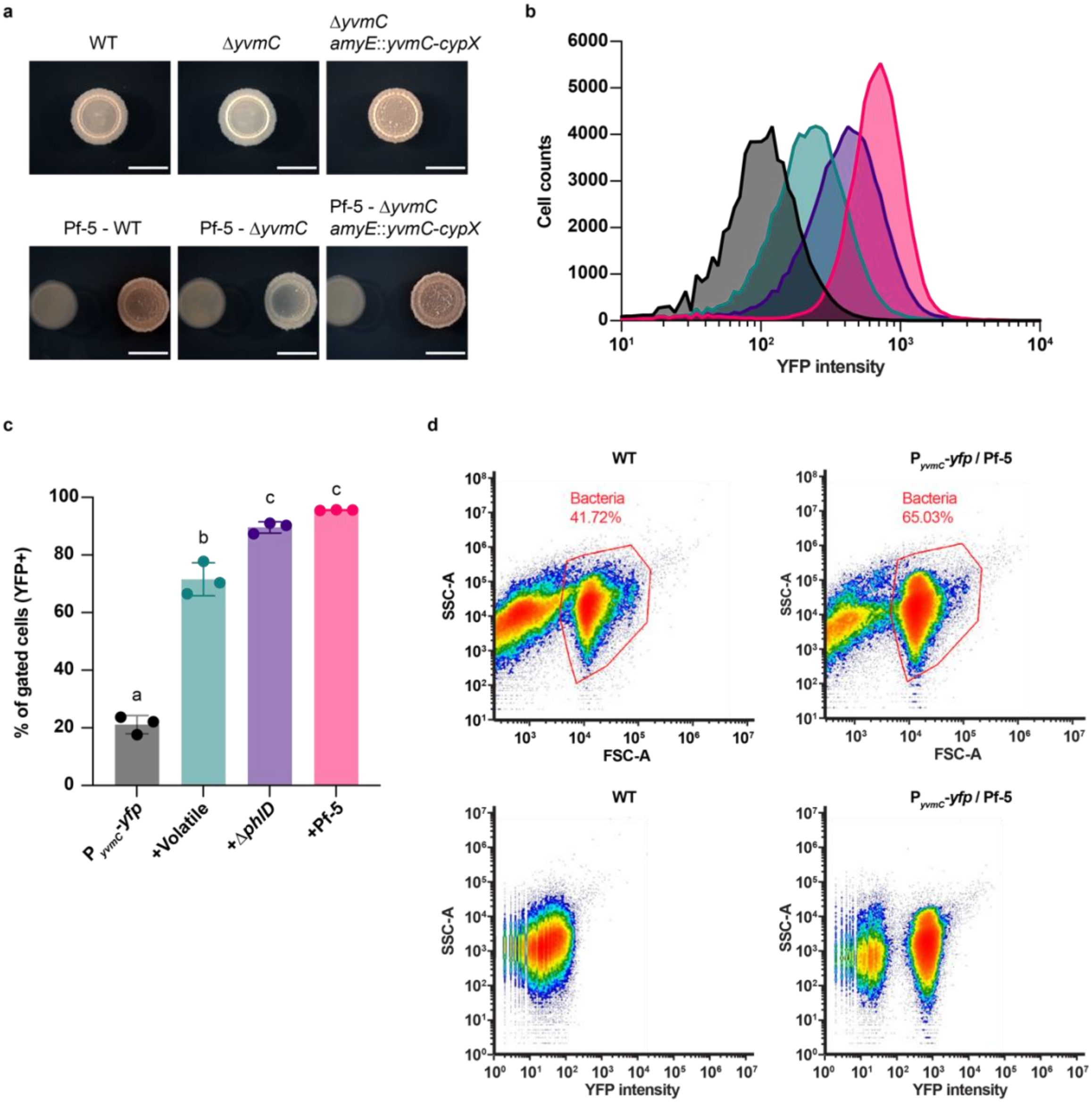

Supplementary Figure 1. *P. protegens* Pf-5 triggers the production of a red pigment in *B. subtilis* which is pulcherriminic acid. a, Representative images of biofilms from *B. subtilis* WT, Δ*yvmC* and Δ*yvmC amyE*::P_{yvmC}-*yvmC-cypX*, in presence and absence of Pf-5 on MS medium after 48 h of growth. **b,** Flow cytometry analysis showing the distribution of fluorescence intensity of YFP-based transcriptional reporter for *yvmC* alone (black), volatile (teal), Δ*phlD* mutant (purple), and Pf-5 (pink). **c,** Histograms showing the mean of cells fluorescent intensity in *B. subtilis* alone (black), volatiles (teal), Δ*phlD* mutant (purple), and with Pf-5 (pink). **d,** Gating strategy used for flow cytometry. *B. subtilis* WT (negative control) and 3610 P_{yvmC}-*yfp* next to Pf-5 (positive control) identify the bacterial population compared to PBS that is always examined before the runs. Data are presented as mean values ± SD, n=3. All experiments were performed in three biological replicates with three technical replicates. Representative experiments and pictures are presented. Different letters indicate statistically significant differences, $P < 0.05$, one-way ANOVA and

Tukey's multiple comparisons test). Source data and exact P value are provided as a Source Data file.

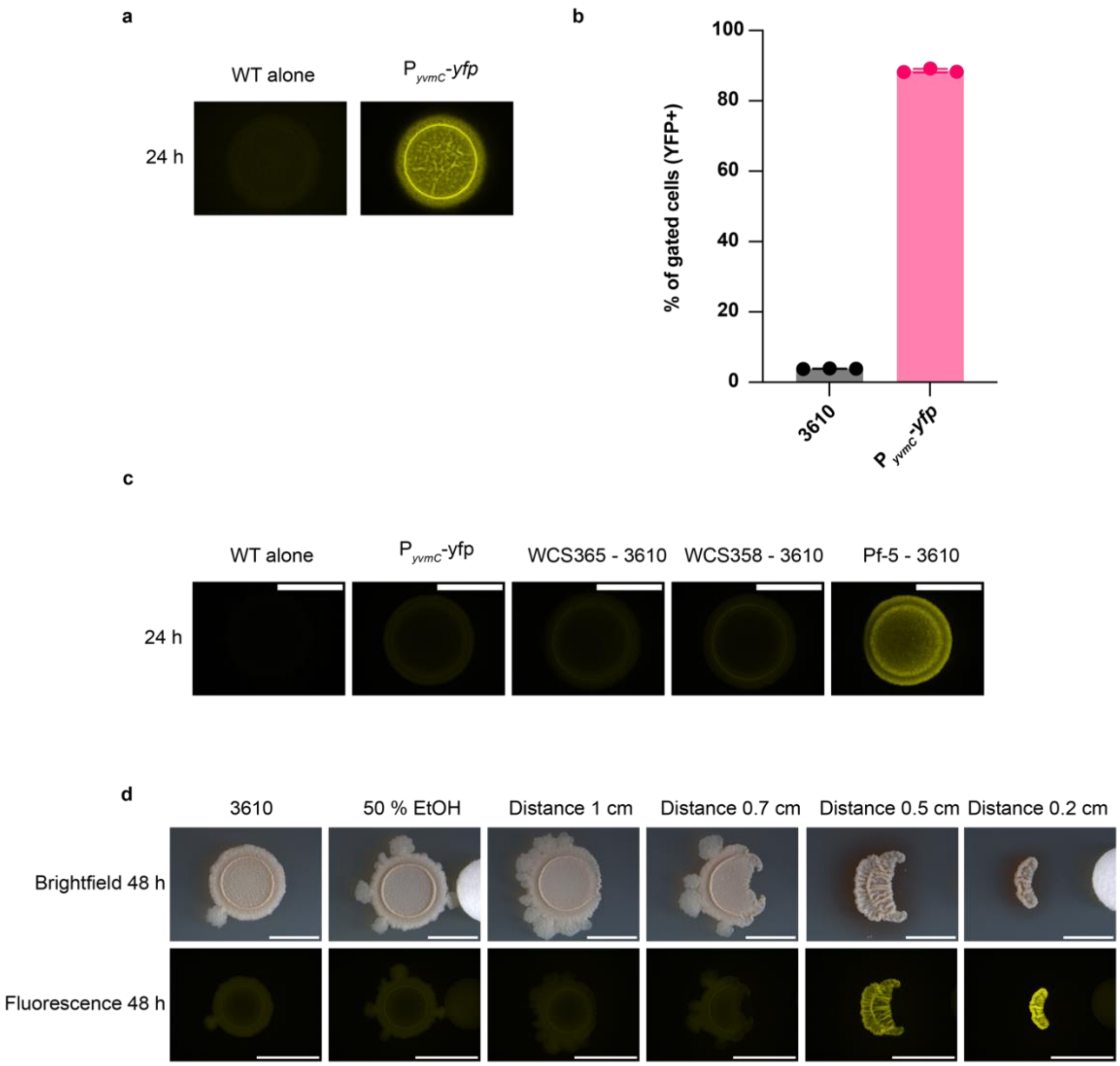

Supplementary Figure 2. Fluorescent microscopy of *B. subtilis* in interspecies competition with WCS365, WCS358, Pf-5, and purified DAPG. **a,** Fluorescent images (false color) of *B. subtilis* (P_{yvmC}-*yfp*) alone on MSgg after 24 h. **b,** Flow cytometry analysis of YFP+ cells (P_{yvmC}-*yfp*) after 24 h of growth on MSgg. Data are presented as mean values ± SD, n=3 **c,** Fluorescent images (false color) of *B. subtilis* WT alone and in presence of WCS365, WCS358, and Pf-5 on MS medium after 24 h of growth. Scale bar, 5 mm. **d,** DAPG was spiked at 0.2 cm, 0.5 cm, 0.7 cm, and 1 cm next to the colonies on a white absorbent paper. Upper row shows brightfield images and the lower row shows fluorescent images (P_{yvmC} - *yfp*). As a control, 50% EtOH was spiked at 0.2 cm from the colony. All experiments were performed in three biological replicates with three technical replicates. Representative experiments and pictures are presented. Scale bar, 5 mm. Source data are provided as a Source Data file.

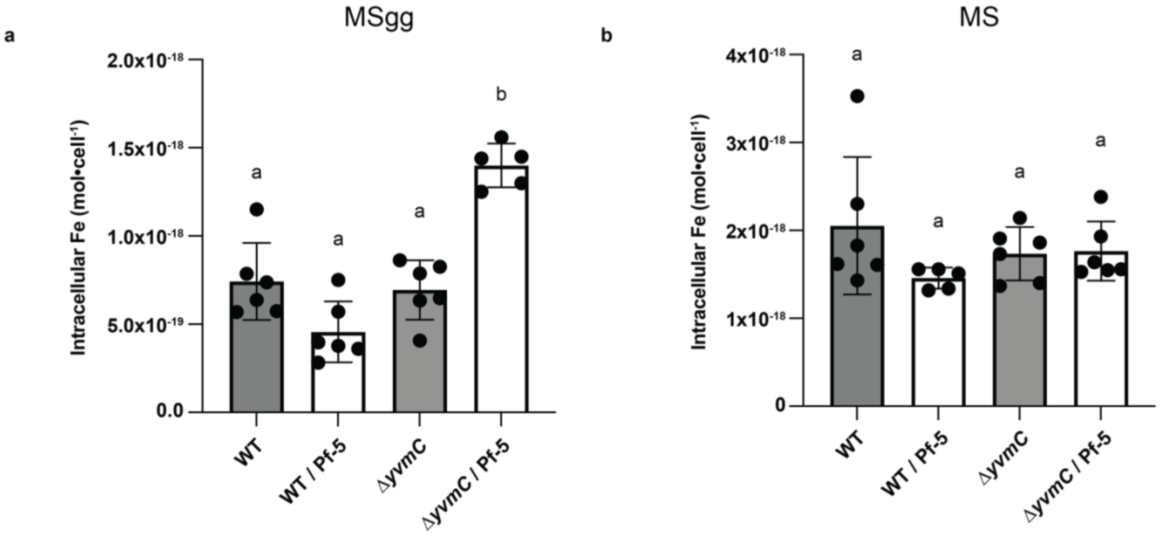

Supplementary Figure 3. Intracellular Fe concentration of WT and Δ*yvmC* with and without Pf-5 a, Intracellular Fe concentration (mol·cell^{-1}) of 48 h colonies formed on MSgg and **b,** on MS medium. Different letters indicate statistically significant differences, P < 0.05, one-way ANOVA and Tukey's multiple comparisons test). Data are presented as mean values ± SD, n=6. Source data and exact P value are provided as a Source Data file.

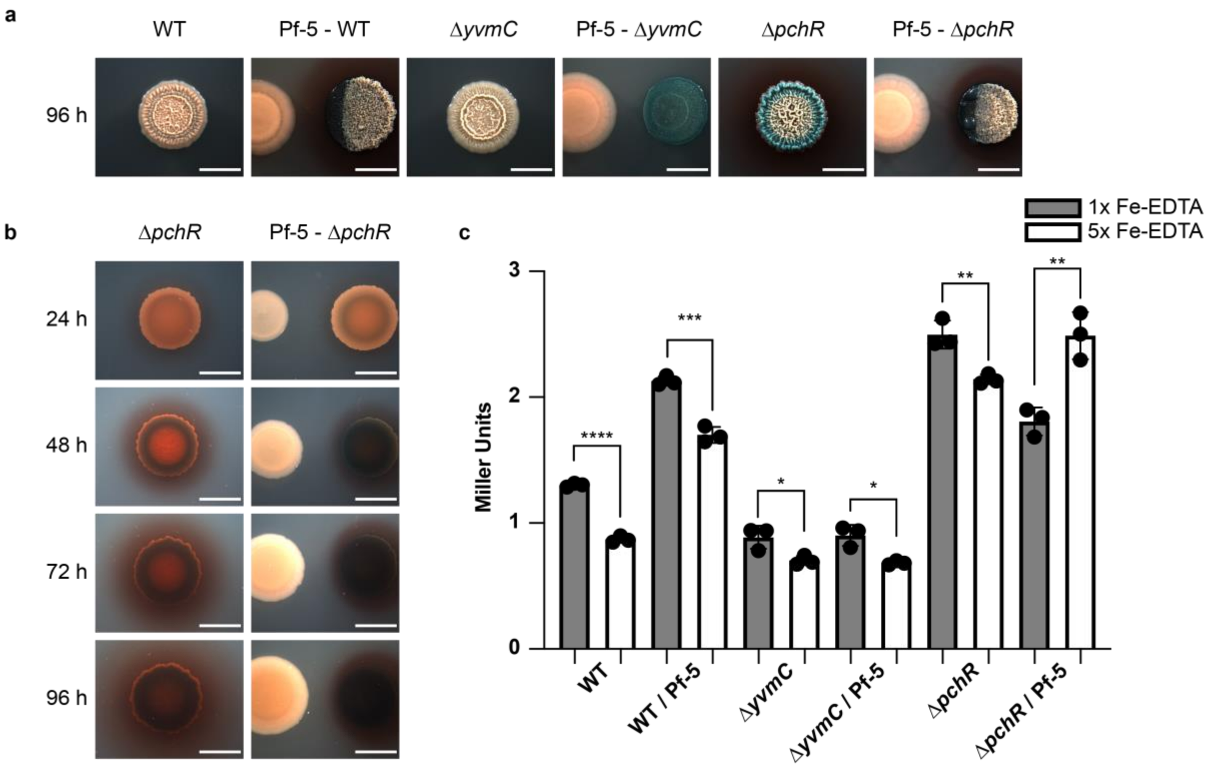

Supplementary Figure 4. Pulcherrimin and Pf-5 induce P$_{dhbA}$-*lacZ* expression in *B. subtilis*. a, Top-view of representative images of biofilms from *B. subtilis* WT, Δ*yvmC*, and Δ*pchR* in interaction with Pf-5. Pictures were taken 96 h on MS supplemented with X-gal at 120 µg/mL. **b,** Below-view of Δ*pchR* mutant alone and in interaction with Pf-5 showing the spatial distribution of *lacZ*-producing cells at 24 h, 48 h, 72 h, and 96 h. Images contrast were adjusted to allow clear visualization. **c,** β-galactosidase activities of WT, Δ*yvmC*, Δ*pchR* harboring the P$_{dhbA}$-*lacZ* reporter alone and in interaction with Pf-5 in MS. Grey bars indicate 1x Fe(III)-EDTA and white bars indicate 5x Fe(III)-EDTA. (*P < 0.05, **P < 0.01. ***P = 5.52 x 10^{-4}, ****P = 1.32 x 10^{-5}, Student's *t* test, two-sided). All experiments were performed in three biological replicates with three technical replicates. Representative experiments and pictures are presented. Data are presented as mean values ± SD, n=3. Scale bar = 5 mm. Source data are provided as a Source Data file.

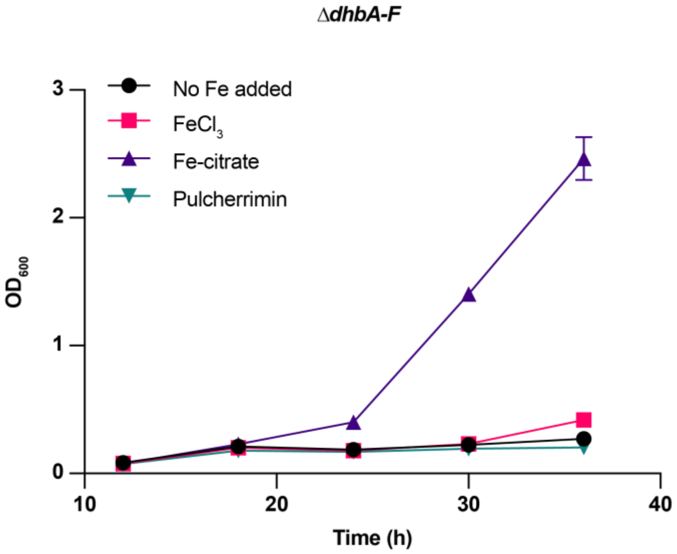

Supplementary Figure 5. *B. subtilis* Δ*dhbA-F* does not grow in presence of pulcherrimin. *B. subtilis* Δ*dhbA-F* growth was monitored (by absorbance at OD_{600}) in MSgg medium. Optical density was measured every 6 h starting at 12 h post-inoculation (12 h, 18 h, 24 h, 30 h, and 36 h) in presence of no added Fe (negative control), 50 µM $FeCl_3$, 50 µM pulcherrimin, and 50 µM Fe-citrate (positive control) as Fe sources. This experiment was performed in three biological replicates with three technical replicates. Data are presented as mean values ± SD, n=3. Source data are provided as a Source Data file.

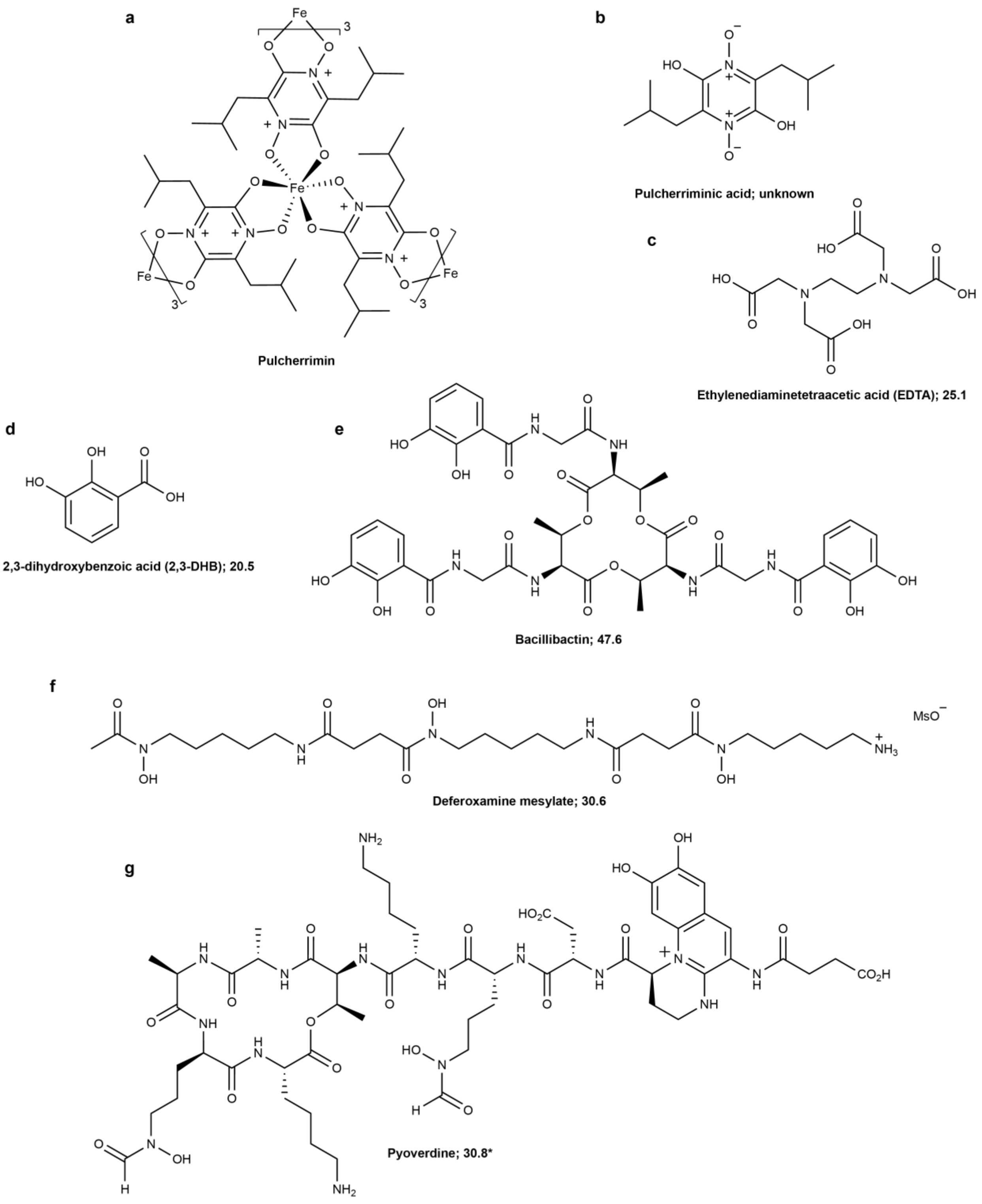

Supplementary Figure 6 Chemical structures of the molecules used throughout this study a, The structure of pulcherrimin **b,** Pulcherriminic acid **c,** EDTA[5] **d,** 2,3-DHB[6] **e,** Bacillibactin[7] **f,** Deferoxamine mesylate[8] **g,** Pyoverdine[9,10]. The binding constant (log *K*) based of Fe(III)-complexes is indicated next to the name of the molecules. * The binding constant is based on *P. aeruginosa*.

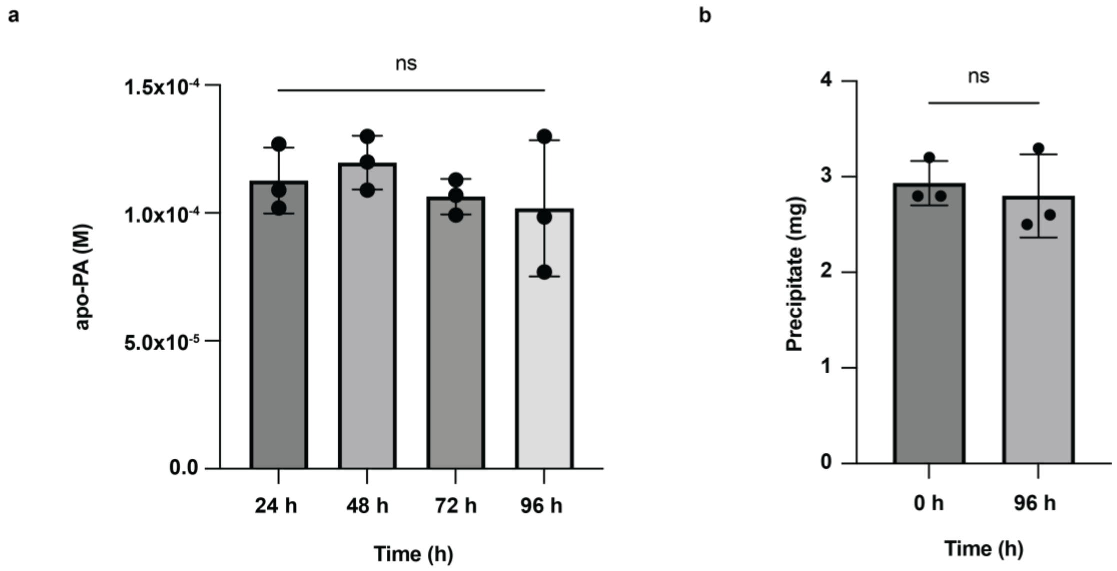

Supplementary Figure 7. The apo-PA and pulcherrimin remain stable over time in aqueous solution. a, Apo-PA (soluble fraction) was quantified at 24 h, 48 h, 72 h, and 96 h without Fe by LC-MS to evaluate the stability over time. (ns; non-significant, one-way ANOVA and Tukey's multiple comparisons test). **b,** The pulcherrimin precipitate was weighted at 0 h and 96 h to evaluate pulcherrimin stability. (ns; non-significant, Student's *t* test, two-sided). Data are presented as mean values ± SD, n=3. Source data are provided as a Source Data file.

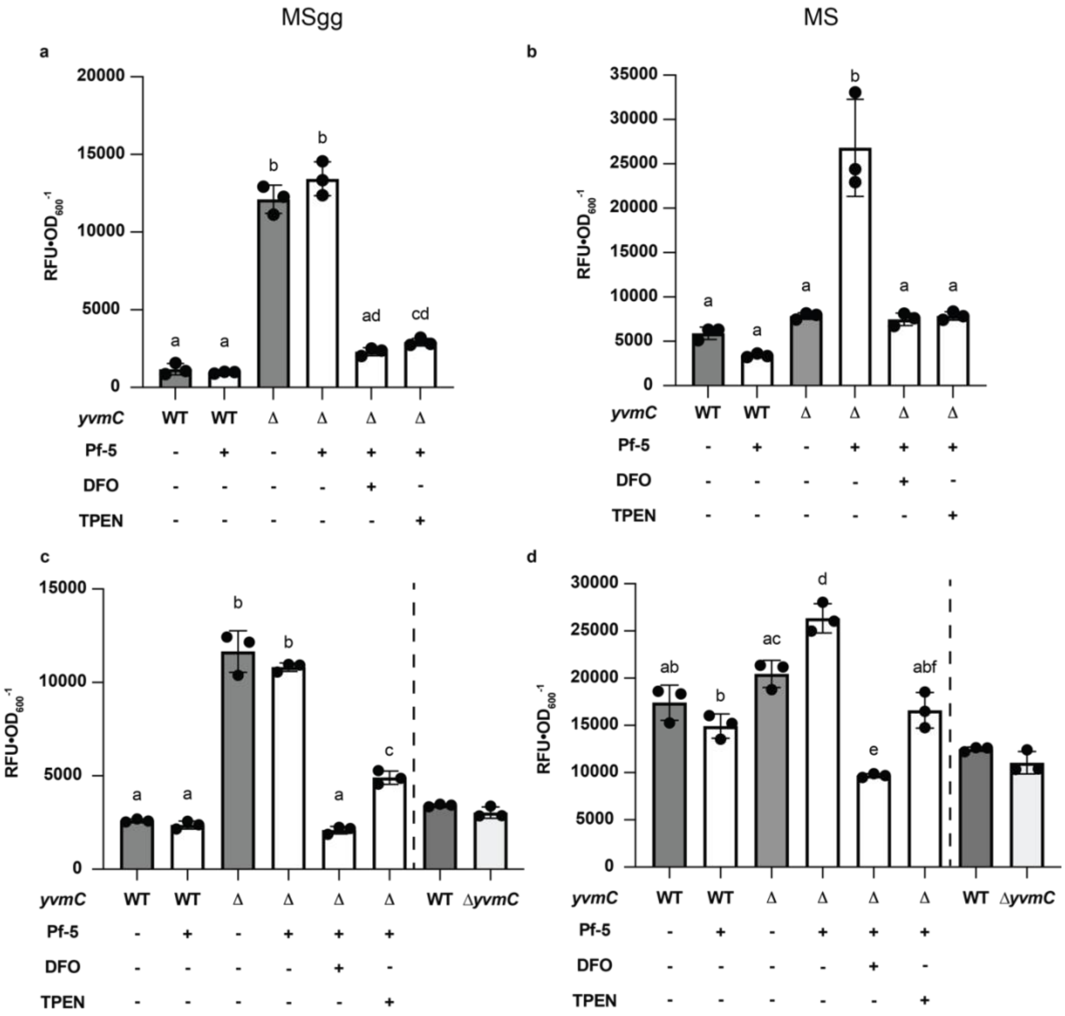

Supplementary Figure 8. Deferoxamine efficiently reduces intracellular oxidative stress compared to the metal chelator TPEN. **a,** Quantification of extracellular ROS using the $DCFH_2$-DA probe in MSgg medium. Fluorescence intensities were normalized on biomass (OD_{600}). DFO indicates that the siderophore deferoxamine mesylate was added to chelate Fe; TPEN indicates that N,N,N′,N′-Tetrakis(2-pyridylmethyl)ethylenediamine was added as metal chelator with a strong zinc affinity. **b,** Same as in a, except that the experiment was conducted in MS medium. **c,** Quantification of intracellular ROS using the $DCFH_2$-DA probe in MSgg medium. Conditions were the same as presented in a, except that a ROS baseline (right of the dashed vertical line) has been added with cells growing exponentially in LB medium. **d,** Same as in c, except that the experiment was performed in MS medium. Experiments was performed in three biological replicates with three technical replicates. Different letters indicate statistically significant differences, $P < 0.05$, one-way ANOVA and Tukey's multiple comparisons test). Data are presented as mean values ± SD, n=3. Source data and exact P value are provided as a Source Data file.

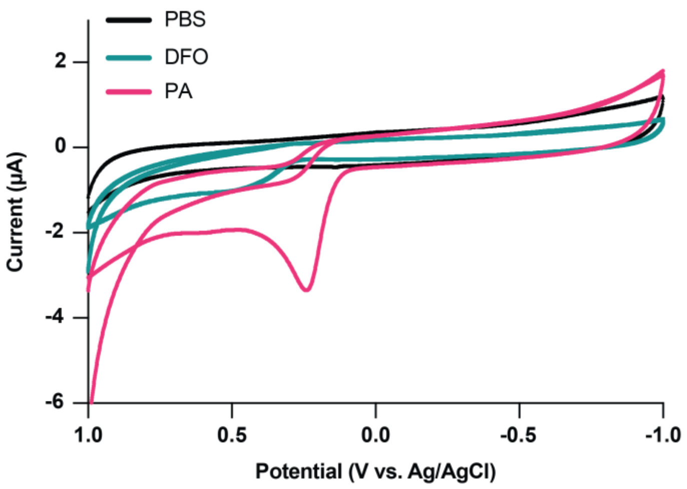

Supplementary Figure 9. Cyclic voltammetry profile curves of Fe-free chelators. Cyclic voltammetry profile curves of DFO (deferoxamine; teal line), PA (pulcherriminic acid; pink line), and PBS (black line).

Supplementary References

1. Usui, I., Lin, D. W., Masuda, T. & Baran, P. S. Convergent Synthesis and Structural Confirmation of Phellodonin and Sarcodonin ε. *Org Lett* **15**, 2080–2083 (2013).

2. Nonappa, Ahonen, K., Lahtinen, M. & Kolehmainen, E. Cyclic dipeptides: catalyst/promoter-free, rapid and environmentally benign cyclization of free amino acids. *Green Chemistry* **13**, 1203–1209 (2011).

3. Dickschat, J. S., Reichenbach, H., Wagner-Döbler, I. & Schulz, S. Novel Pyrazines from the Myxobacterium Chondromyces crocatus and Marine Bacteria. *European J Org Chem* **2005**, 4141–4153 (2005).

4. Koo, B. *et al.* Libraries for *Bacillus subtilis*. *Cell Syst* **4**, 291-305.e7. (2017).

5. Allen, H. E. & Chen, P.-H. Remediation of metal contaminated soil by EDTA incorporating electrochemical recovery of metal and EDTA. *Environmental Progress* **12**, 284–293 (1993).

6. Avdeef, A., Sofen, S. R., Bregante, T. L. & Raymond, K. N. Coordination chemistry of microbial iron transport compounds. 9. Stability constants for catechol models of enterobactin. *J Am Chem Soc* **100**, 5362–5370 (1978).

7. Dertz, E. A., Xu, J., Stintzi, A. & Raymond, K. N. Bacillibactin-Mediated Iron Transport in Bacillus subtilis. *J Am Chem Soc* **128**, 22–23 (2006).

8. Bellotti, D. & Remelli, M. Deferoxamine B: A Natural, Excellent and Versatile Metal Chelator. *Molecules* **26,** 3265 (2021).

9. L., H. S. *et al.* Ferric-Pyoverdine Recognition by Fpv Outer Membrane Proteins of Pseudomonas protegens Pf-5. *J Bacteriol* **195**, 765–776 (2013).

10. Albrecht-Gary, A.-M., Blanc, S., Rochel, N., Ocaktan, A. Z. & Abdallah, M. A. Bacterial Iron Transport: Coordination Properties of Pyoverdin PaA, a Peptidic Siderophore of Pseudomonas aeruginosa. *Inorg Chem* **33**, 6391–6402 (1994).
