# Supplementary material for: Pulcherriminic acid modulates iron availability and protects against oxidative stress during microbial interactions
Source: Nat Commun. 2023 May 3;14:2536. doi: 10.1038/s41467-023-38222-0 (PMC10156857; doi:10.1038/s41467-023-38222-0)
Supplement: Supplementary file 3 — Reporting Summary [file 41467_2023_38222_MOESM3_ESM.pdf]

nature portfolio

Corresponding author(s): Charron-Lamoureux V & Beauregard PB

Last updated by author(s): Mar 29, 2023

Reporting Summary

Nature Portfolio wishes to improve the reproducibility of the work that we publish. This form provides structure for consistency and transparency in reporting. For further information on Nature Portfolio policies, see our Editorial Policies and the Editorial Policy Checklist.

Statistics

For all statistical analyses, confirm that the following items are present in the figure legend, table legend, main text, or Methods section.

n/a	Confirmed	
☐	☒	The exact sample size (*n*) for each experimental group/condition, given as a discrete number and unit of measurement
☐	☒	A statement on whether measurements were taken from distinct samples or whether the same sample was measured repeatedly
☐	☒	The statistical test(s) used AND whether they are one- or two-sided<br>*Only common tests should be described solely by name; describe more complex techniques in the Methods section.*
☒	☐	A description of all covariates tested
☒	☐	A description of any assumptions or corrections, such as tests of normality and adjustment for multiple comparisons
☐	☒	A full description of the statistical parameters including central tendency (e.g. means) or other basic estimates (e.g. regression coefficient) AND variation (e.g. standard deviation) or associated estimates of uncertainty (e.g. confidence intervals)
☐	☒	For null hypothesis testing, the test statistic (e.g. *F*, *t*, *r*) with confidence intervals, effect sizes, degrees of freedom and *P* value noted<br>*Give P values as exact values whenever suitable.*
☒	☐	For Bayesian analysis, information on the choice of priors and Markov chain Monte Carlo settings
☒	☐	For hierarchical and complex designs, identification of the appropriate level for tests and full reporting of outcomes
☒	☐	Estimates of effect sizes (e.g. Cohen's *d*, Pearson's *r*), indicating how they were calculated

Our web collection on statistics for biologists contains articles on many of the points above.

Software and code

Policy information about availability of computer code

Data collection	FACS; BD CSampler plus version 1.0.23.1 and BD FACS Sorftware 1.2.0.142. TECAN (serial number: 2103000038); SparkControl version 3.1
Data analysis	Statistical analysis was performed with GraphPad Prism 9 version 9.4.0. Fiji (ImageJ1; version 2.9.0/ 1.53t) was used for image analysis. For flow cytometry, the BD software was used (for versions, see data collection)

For manuscripts utilizing custom algorithms or software that are central to the research but not yet described in published literature, software must be made available to editors and reviewers. We strongly encourage code deposition in a community repository (e.g. GitHub). See the Nature Portfolio guidelines for submitting code & software for further information.

Data

Policy information about availability of data

All manuscripts must include a data availability statement. This statement should provide the following information, where applicable:
- Accession codes, unique identifiers, or web links for publicly available datasets
- A description of any restrictions on data availability
- For clinical datasets or third party data, please ensure that the statement adheres to our policy

The source data for Figs. 1-6, Supplementary Figs. 1-9 are provided in the Source Data file.

Human research participants

Policy information about studies involving human research participants and Sex and Gender in Research.

Reporting on sex and gender	N/A
Population characteristics	N/A
Recruitment	N/A
Ethics oversight	N/A

Note that full information on the approval of the study protocol must also be provided in the manuscript.

Field-specific reporting

Please select the one below that is the best fit for your research. If you are not sure, read the appropriate sections before making your selection.

[x] Life sciences [ ] Behavioural & social sciences [ ] Ecological, evolutionary & environmental sciences

For a reference copy of the document with all sections, see nature.com/documents/nr-reporting-summary-flat.pdf

Life sciences study design

All studies must disclose on these points even when the disclosure is negative.

Sample size	No statistical methods were used to predetermine sample size. Samples size ranges from 3 to 6, depending on the assays with at least three biological replicates except for the ICP-MS and dissociation kinetics where n=3 or n=6 were used as a sample size. Most of experiments were conducted with three technical replicates which is sufficient to capture the mean and perform appropriate statistical analysis and with three biological replicates which confirmed reproducibility.
Data exclusions	No data were excluded, except two values in Supplementary Figure 3. See Data Source for Fig. S3. The values qualified as an outlier based on the Z score (q-test). Outliers were excluded from mean and SD calculations.
Replication	Experiments were performed at least in three independents biological replicates. All replicates were consistent and confirmed.
Randomization	All Experiments that started with a period of incubation where randomly distributed in the incubation process.
Blinding	Experiments were conducted in blinded ways with two different persons to confirm reproducibility and reduced bias. Experiments to determine oxidative stress level (fig. 6) were conducted by two persons as well as Fig. 2, and Supplementary Figure 2.

Reporting for specific materials, systems and methods

We require information from authors about some types of materials, experimental systems and methods used in many studies. Here, indicate whether each material, system or method listed is relevant to your study. If you are not sure if a list item applies to your research, read the appropriate section before selecting a response.

Materials & experimental systems

n/a	Involved in the study	
[x]	[ ]	Antibodies
[x]	[ ]	Eukaryotic cell lines
[x]	[ ]	Palaeontology and archaeology
[x]	[ ]	Animals and other organisms
[x]	[ ]	Clinical data
[x]	[ ]	Dual use research of concern

Methods

n/a	Involved in the study	
[x]	[ ]	ChIP-seq
[ ]	[x]	Flow cytometry
[x]	[ ]	MRI-based neuroimaging

Flow Cytometry

Plots

Confirm that:

- [x] The axis labels state the marker and fluorochrome used (e.g. CD4-FITC).
- [x] The axis scales are clearly visible. Include numbers along axes only for bottom left plot of group (a 'group' is an analysis of identical markers).
- [x] All plots are contour plots with outliers or pseudocolor plots.
- [x] A numerical value for number of cells or percentage (with statistics) is provided.

Methodology

Sample preparation	Bacterial cells grown for 24 h (biofilms) were removed from the agar surface with a P200 tip and placed into an Eppendorf tube containing 1 mL of PBS. Cells were sonicated with an amplitude of 30% between 30-60 s without pause until homogeneity, then centrifuged at 13800 x g for 2 min before adding 200 μL of paraformaldehyde 4%. Cells were fixed for 7 min and washed twice with PBS before analysis
Instrument	BD FACSJazz (2015, JZ6554902601) and BD Accuri C6 plus (2017, AC66051710456)
Software	BD CSampler plus version 1.0.23.1 and BD FACS Sortware 1.2.0.142
Cell population abundance	BD software was set to 100000 events and each sample stop after reaching that number of events. The gating which include bacteria range between 59000 events (bacteria) and 70000 events (bacteria) and the fluorescence intensity was measured based on the bacterial gate.
Gating strategy	FSC/SSC gates used for the flow cytometry experiment are provided in Supplementary Figure 1. A negative control (YFP-), a positive control (YFP+) and a PBS control (not shown) was used to create FSC/SSC gates.

- [x] Tick this box to confirm that a figure exemplifying the gating strategy is provided in the Supplementary Information.
